# Assessment of hepatitis B and tetanus toxoid vaccination coverage and associated factors among healthcare workers at Kabul University of Medical Sciences affiliated hospitals in Afghanistan

**DOI:** 10.1186/s12889-026-27775-6

**Published:** 2026-07-03

**Authors:** Parvin Golzareh, Shafiqullah Shahim, Nargis Usmani, Erin M. Mann, Ajmal Sabawoon, Mohammad Sedique Yousofzai

**Affiliations:** 1 Advanced Midwifery Skills Department, School of Midwifery, Kabul University of Medical Sciences “Abu Ali Ibn Sina”, Kabul, Afghanistan; 2 Health Management and Policy Department, School of Public Health, Kabul University of Medical Sciences “Abu Ali Ibn Sina”, Kabul, Afghanistan; 3 Normal Midwifery Department, School of Midwifery, Kabul University of Medical Sciences “Abu Ali Ibn Sina”, Kabul, Afghanistan; 4 Division of Infectious Diseases and International Medicine, Department of Medicine, University of Minnesota, 420 Delaware St SE, Minneapolis, MN 55455 United States of America; 5 Department of Epidemiology, Mailman School of Public Health, Columbia University, New York City, United States of America; 6 Infectious Disease Department, School of Medicine, Kabul University of Medical Sciences “Abu Ali Ibn Sina”, Kabul, Afghanistan

**Keywords:** Hepatitis B vaccine, Tetanus toxoid vaccine, Healthcare workers, Afghanistan

## Abstract

**Objective:**

Hepatitis B and tetanus remain significant public health challenges in Afghanistan, a developing country where vaccination coverage is suboptimal. Healthcare workers are particularly at risk due to their exposure to infected blood and bodily fluids, and they play a vital role as health role models; however, their vaccination rates for hepatitis B and tetanus are often inadequate, risking both their health and potential transmission to patients. This study aimed to assess the vaccination coverage and associated factors among health personnel in teaching hospitals affiliated with Kabul University of Medical Sciences, providing insights to inform strategies for improving immunization practices and occupational safety.

**Methods:**

A cross-sectional study was conducted at three teaching hospitals affiliated with Kabul University of Medical Sciences. The study sample included medical personnel (physicians, nurses, midwives, laboratory technicians, and other clinical staff) actively engaged in patient care with direct exposure to blood or bodily secretions. Demographic and vaccination information were gathered from them using questionnaires. Descriptive statistics and logistic regression were performed using Stata version 18.0.

**Results:**

A total of 284 professional staff members from the three teaching hospitals of Kabul University of Medical Sciences participated in this study from 20 December 2024 to 20 March 2025. The majority were male (52.8%), with ages ranging from 19 to 62 years. Hepatitis B vaccination coverage was 57.8% (full) and 67.3% (partial or full); tetanus toxoid vaccination coverage was 45.5% (full) and 49.3% (partial or full). Factors associated with hepatitis B vaccination included profession and work experience. Tetanus vaccination was strongly associated with gender and profession, with a trend toward higher odds among those with higher income. The most commonly cited reason for non-vaccination was lack of access (48.4%), including geographical barriers, unavailability of vaccines at health facilities, and cost.

**Conclusion:**

Hepatitis B and tetanus vaccination coverage among healthcare workers in Kabul was suboptimal but comparable to that reported in other developing countries, with distinct factors driving uptake for each vaccine. Despite near-universal positive attitudes toward vaccination, the primary barrier was lack of access, highlighting systemic gaps in occupational health services. Institutional vaccination programs with free, on-site access are needed to protect healthcare workers and their patients. Further studies with larger samples across diverse settings are needed to confirm these findings.

**Supplementary Information:**

The online version contains supplementary material available at https://doi.org/10.1186/s12889-026-27775-6.

## Introduction

Hepatitis B virus (HBV) and tetanus are two vaccine-preventable diseases that are still major public health issues in developing countries, including Afghanistan [1, 2]. Hepatitis B is a viral infection affecting the liver that can manifest as either a short-term or chronic condition, with chronic infection placing individuals at risk of death from cirrhosis or liver cancer. According to World Health Organization (WHO), 254 million people were living with chronic hepatitis B infection in 2022, with 1.2 million new infections each year. HBV prevalence in Afghanistan is estimated at 1.8–2% in the general population [3]. Tetanus, also known as lockjaw, is a serious and potentially fatal infection caused by the bacterium *Clostridium tetani*, preventable through immunization with tetanus toxoid-containing vaccine. Globally, an estimated 50,000 deaths from tetanus occur annually, with the highest burden in low-income countries [4]. Tetanus burden in Afghanistan is difficult to quantify due to the absence of a national disease registry; however, case fatality rates have been reported at approximately 50% [5]. 

The WHO and United States Centers for Disease Control and Prevention (CDC) recommend the hepatitis B vaccine for all newborns, children, and unvaccinated individuals under 19, as well as adults aged 19–59 and at-risk adults aged 60 and older, administered in 2–3 doses over 6 months depending on the brand [6–8]. For tetanus, the DPT vaccine is recommended for children, adolescents, and adults, with a booster dose every 10 years, or after 5 years in the event of a severe or contaminated wound or burn [9]. 

Healthcare workers (HCWs) face a significantly greater risk of vaccine-preventable infections due to their direct contact with patients and exposure to blood, bodily fluids, and contaminated instruments. HCWs in low-income countries are particularly vulnerable to HBV, where limited resources and safety protocols can exacerbate the risk of transmission [10]. HCWs are also at elevated risk of tetanus through occupational exposure to wounds and contaminated instruments, a risk heightened in settings with limited infection prevention infrastructure. Critically, the risk of HBV is not one-directional; an HCW infected with HBV can potentially transmit the virus to their patients, creating a dangerous cycle of transmission within healthcare settings. These risks highlight the urgent need for comprehensive vaccination for all health professionals [11]. 

Vaccine delivery in Afghanistan is primarily managed by public health facilities and is free of charge for children and mothers. Through the Basic Package of Health Services (BPHS) program, accessibility to vaccines has been increased even in rural areas; however, there is no formal vaccination program or policy for adult healthcare workers, and vaccination remains at the discretion of the individual. The Multiple Indicator Cluster Survey (MICS) conducted by UNICEF for the period of 2022–2023 revealed that only 36.6% of children aged 12–23 months received basic immunization, while 3rd dose coverage of hepatitis B and tetanus vaccination among children under five was 59% overall [12, 13]. No data on tetanus or hepatitis B vaccination coverage among the adult or general population in Afghanistan are available. Regarding hepatitis B vaccination coverage among health personnel, only one study conducted in 2018–2019 found that 56.37% of health workers had received the first dose of the HBV vaccine, and only 6.77% had completed all three doses [14]. No study has been conducted regarding tetanus vaccination coverage among health workers in Afghanistan.

Despite the importance of vaccination, there are no protocols or requirements for healthcare worker vaccination in Afghanistan as a condition of employment, and vaccination remains completely at the discretion of healthcare workers. Given that Kabul University of Medical Sciences (KUMS) and its affiliated teaching hospitals are the main training centers for health personnel, they serve as a model for other health facilities in terms of knowledge, attitudes, and the implementation of preventive health programs, including vaccination.

The aim of this study was to examine the coverage of hepatitis B and tetanus vaccination and the associated factors among health personnel working in teaching hospitals affiliated with KUMS. The results of this study may identify the need for changes in behavior and practices regarding hepatitis B and tetanus vaccination among health personnel in teaching hospitals.

## Material & methods

A cross-sectional study was conducted from December 20, 2024, to March 20, 2025, across three KUMS-affiliated teaching hospitals in Kabul, Afghanistan. The study sample included medical personnel (physicians, nurses, midwives, laboratory technicians, and other clinical staff) actively engaged in patient care with direct exposure to blood or bodily secretions. Participants were selected proportionally from each professional category using stratified random sampling.

### Sample size calculation

Sample size was calculated using Cochran’s formula with a finite population correction. The initial sample size was determined as n₀ = Z²p(1 − p)/E² = (1.96)²(0.5)(0.5)/(0.05)² = 385, where Z = 1.96 (for a 95% confidence level), *p* = 0.5 (assumed proportion), and E = 0.05 (margin of error). The finite population correction was then applied based on the total healthcare worker population across the three hospitals (*N* = 764): n = n₀/(1 + (n₀−1)/N) = 385/(1 + 384/764) = 256.22, rounded to 257. Accounting for a 10% non-response rate, the target sample size was 283 (257 × 1.10 = 282.7, rounded to 283). Participants were selected using stratified random sampling, with strata defined by health worker category and gender, and proportions determined by subgroup size.

### Sampling method

Stratified random sampling was employed. A comprehensive list of eligible healthcare personnel from KUMS-affiliated hospitals was compiled, including physicians, nurses, midwives, laboratory technicians, and X-ray technicians. Strata were defined by health worker category and gender, and participants were proportionally allocated to each stratum based on subgroup size relative to the total workforce, ensuring the sample reflected the composition of the target population. Within each stratum, individuals were randomly selected using simple random sampling. The sample size was not inflated to maintain statistical power within individual strata, as the primary research questions were designed for aggregate-level analyses.

### Inclusion and exclusion criteria

Eligibility for this study was strictly limited to healthcare personnel with direct patient contact and a minimum of six months of hospital work experience. Direct patient contact was defined as routine involvement in clinical care with direct exposure to patients’ blood or bodily fluids, including physical examination and treatment, face-to-face interviews, direct therapeutic interventions, and bedside care. Consequently, individuals were excluded if they had not engaged in direct patient care within the preceding two years. All eligible candidates were required to provide signed informed consent prior to their enrollment in the study.

### Data collection and management

Data were collected using a structured, self-administered paper-based questionnaire developed by the study team; an English-language version of the questionnaire is provided (Supplementary File). The questionnaire captured demographic information, health background, vaccination history, and participants’ beliefs about vaccination and reasons for non-vaccination. Questionnaires were reviewed immediately following administration to identify and address missing data. Following data cleaning, the information was entered into a computer database and subsequently analyzed. Ethical approval was obtained from the KUMS Institutional Review Board prior to data collection (Protocol No. 13, December 7, 2024). Informed consent to participate in the study was obtained from all participants. Participant anonymity was strictly maintained, participation was voluntary, and no harms were incurred.

### Study variables

The primary outcome variables were hepatitis B and tetanus toxoid vaccination status. For bivariate and regression analyses, vaccination status was dichotomized as vaccinated (combining partially and fully vaccinated participants) versus not vaccinated due to limited frequencies in the partial vaccination category. Full vaccination rates were also calculated for descriptive purposes. Full hepatitis B vaccination was defined as receipt of three doses per WHO recommendations. Full tetanus toxoid vaccination was defined as receipt of five doses, consistent with the WHO-recommended schedule for prevention of maternal and neonatal tetanus, which serves as the basis for Afghanistan’s national routine immunization program.

Predictor variables included in the regression models were age, gender, marital status, monthly income, profession, and work experience. Additional variables collected and examined in bivariate analyses included educational level; additional variables collected for descriptive purposes included department, place of vaccination, and participants’ beliefs regarding vaccination (assessed as a single item indicating willingness to receive the vaccine).

Age was categorized into four groups (19–24, 25–34, 35–44, and 45–62 years) based on the study population’s age distribution. Work experience was categorized as 1–5 years and six or more years, as all participants met the minimum six-month eligibility requirement and no participants reported less than one year of experience.

Profession was categorized as physicians, midwives/nurses, and other health professionals (laboratory technicians, X-ray technicians, and medical assistants). Nurses and midwives were combined into a single category due to their overlapping scopes of practice and similar clinical roles within Afghan hospital settings, as well as small subgroup sample sizes.

### Statistical analysis

Descriptive statistics were used to summarize participant characteristics, with frequencies and percentages reported for categorical variables and means and standard deviations for continuous variables. Bivariate associations between vaccination status and participant characteristics were assessed using design-based Pearson chi-square tests, with results reported as survey-adjusted F-statistics to account for the stratified sampling design. A p-value of < 0.05 was considered statistically significant.

Variables were included in the multivariable models based on their theoretical relevance to vaccination uptake and results of the bivariate analyses. The final adjusted models included age, gender, marital status, work experience, profession, and monthly income. Educational level was excluded due to evidence of multicollinearity with profession. Separate binary logistic regression models were constructed for hepatitis B and tetanus vaccination [15]. To account for the stratified sampling design, logistic regression analyses were conducted using survey-weighted methods. Multicollinearity among predictor variables was assessed using variance inflation factors (VIF). Both crude and adjusted odds ratios (OR) with 95% confidence intervals (CI) were calculated. All analyses were performed using Stata version 18.0.

## Results

### Participant characteristics

This study included 284 professional staff members (Table 1) from the three teaching hospitals of KUMS: Aliabad Teaching Hospital, Maiwand Teaching Hospital, and Shahrara Teaching Hospital. The majority of participants were male (52.8%), with ages ranging from 19 to 62 years and an average age of 32. Over half of the participants (63.4%) were married. In terms of education, the largest group were Grade 14 graduates (32.4%), followed by a Bachelor’s degree (31.0%), MD (25.0%), specialist degree (7.4%), and Master’s degree (4.2%). Participants had an average of 7 years of work experience (SD = 7.5), with a range of 1 to 35 years. Regarding job categories, physicians constituted the largest group (37.3%), followed by nurses and midwives (35.9%). Laboratory technicians, X-ray technicians, and medical assistants together accounted for approximately 27% of the participants. Participants were drawn from various departments across the teaching hospitals. The surgery department contributed the highest number of participants (29.6%), followed by internal medicine (15.8%), while the urology department contributed the lowest (1.1%). These sample proportions match the actual proportion of these professions across KUMS-affiliated hospitals.

**Table 1:** Sociodemographic and professional characteristics of healthcare workers at three KUMS-affiliated teaching hospitals in Kabul, Afghanistan (*N* = 284)

Variable	Frequency	Percentage
Age		
19–24 years	40	14.08
25–34 years	162	57.04
35–44 years	50	17.61
45–62 years	32	11.27
Gender		
Male	150	52.82
Female	134	47.18
Marital Status		
Single	104	36.62
Married	180	63.38
Educational Level		
Grade 14	92	32.39
Bachelor	88	30.99
Master	12	4.23
MD	71	25.00
Specialist	21	7.39
Total Work Experience		
1–5 years	172	60.56
≥ 6 years	112	39.44
Profession		
Physicians	106	37.32
Midwives/Nurses	102	35.92
Other Health Professions	76	26.76
Monthly Income		
Less than 10,000 AFs	48	16.90
10,000–20,000 AFs	186	65.49
More than 20,000 AFs	50	17.61
Place of HBV Vaccination		
Governmental Facilities	65	34.03
Private Facilities	126	65.97
Place of Tetanus Vaccination		
Governmental Facilities	129	92.14
Private Facilities	11	7.86

### Hepatitis B vaccination coverage

The proportion of participants who had received three doses of hepatitis B vaccination was 57.8%. Overall, 67.3% of participants were vaccinated (partially or fully) against hepatitis B. Education level was significantly associated with vaccination (*p* = 0.023), with vaccination coverage increasing progressively from 51.1% among Grade 14 graduates to 90.5% among specialists. Total work experience was also a significant factor (*p* = 0.008); a higher proportion of healthcare workers with six or more years of experience were vaccinated (76.8%) compared to those with 1–5 years of experience (61.1%). Although not statistically significant, higher vaccination rates were observed among older age groups (*p* = 0.211), females (69.4% vs. 65.3% in males), married individuals (70.0% vs. 62.5% in singles), and those with a monthly income above 20,000 AFs (74.0%). No significant differences were found for profession or monthly income (Table 2). Among those vaccinated, 66.0% received their vaccine at a private facility and 34.0% at a governmental facility (Table 1).

**Table 2:** Bivariate analysis of factors associated with hepatitis B vaccination among healthcare workers at Kabul University of Medical Sciences affiliated hospitals (*N* = 284)

Variable	*N*	Non-vaccinated	Vaccinated	*P*-value
Age				
19–24 years	40	45.00	55.00	0.211
25–34 years	162	34.57	65.44	
35–44 years	50	22.00	78.00	
45–62 years	32	25.00	75.00	
Gender				
Male	150	34.67	65.33	0.590
Female	134	30.60	69.40	
Marital Status				
Single	104	37.50	62.50	0.357
Married	180	30.00	70.00	
Educational Level				
Grade 14	92	48.91	51.08	0.023
Bachelor	88	26.14	73.87	
Master	12	16.67	83.33	
MD	71	29.58	70.42	
Specialist	21	9.52	90.48	
Total Work Experience				
1–5 years	172	38.95	61.05	0.008
≥ 6 years	112	23.21	76.78	
Profession				
Physicians	106	27.36	72.64	0.107
Midwives/Nurses	102	40.20	59.80	
Other Health Professions	76	30.26	69.74	
Monthly Income				
Less than 10,000 AFs	48	29.17	70.83	0.271
10,000–20,000 AFs	186	35.48	64.52	
More than 20,000 AFs	50	26.00	74.00	

*P-*values are based on design-based Pearson chi-square tests, reported as survey-adjusted F-statistics to account for the stratified sampling design

### Tetanus vaccination coverage

The proportion of participants who had received five doses of tetanus toxoid vaccination was 45.5%. Overall, 49.3% of participants were vaccinated (partially or fully) against tetanus. Gender was the only factor significantly associated with tetanus vaccination status (*p* = 0.001). A strikingly higher proportion of female healthcare workers were vaccinated (82.8%) compared to their male counterparts (19.3%). Although not statistically significant, some notable patterns were observed across other variables. Vaccination coverage tended to be higher among younger age groups, with 65.0% of those aged 19–24 years vaccinated compared to only 30.0% in the 35–44 age group and 37.5% in the 45–62 age group (*p* = 0.217). Single individuals showed higher vaccination coverage (59.6%) than married participants (43.3%), though this difference was not significant (*p* = 0.159). Among professional groups, midwives and nurses had the highest vaccination rate (63.7%), while other health professions had the lowest (40.8%), yet these differences did not reach statistical significance (*p* = 0.783). No significant associations were found for educational level, total work experience, or monthly income (Table 3).

**Table 3:** Bivariate analysis of factors associated with tetanus vaccination among healthcare workers at Kabul University of Medical Sciences affiliated hospitals (*N* = 284)

Variable		*N*	Non-vaccinated			Vaccinated		*P*-value
Age								
19–24 years		40	35.00			65.00		0.217
25–34 years		162	46.30			53.71		
35–44 years		50	70.00			30.00		
45–62 years		32	62.50			37.50		
Gender								
Male		150	80.67			19.33		0.001
Female		134	17.16			82.83		
Marital Status								
Single		104	40.38			59.62		0.159
Married		180	56.67			43.33		
Educational Level								
Grade 14		92	47.83			52.17		0.750
Bachelor		88	50.00			50.00		
Master		12	41.67			58.34		
MD		71	59.15			40.85		
Specialist		21	42.86			57.15		
Total Work Experience								
1–5 years		172	45.93			54.07		0.393
≥ 6 years		112	58.04			41.96		
Profession								
Physicians		106	58.49			41.51		0.783
Midwives/Nurses		102	36.27			63.73		
Other Health Professions		76	59.21			40.79		
Monthly Income								
Less than 10,000 AFs		48	60.42			39.58		0.558
10,000–20,000 AFs		186	46.77			53.23		
More than 20,000 AFs		50	56.00			44.00		

*P-*values are based on design-based Pearson chi-square tests, reported as survey-adjusted F-statistics to account for the stratified sampling design

### Reasons for not vaccinating

**Fig. 1:**
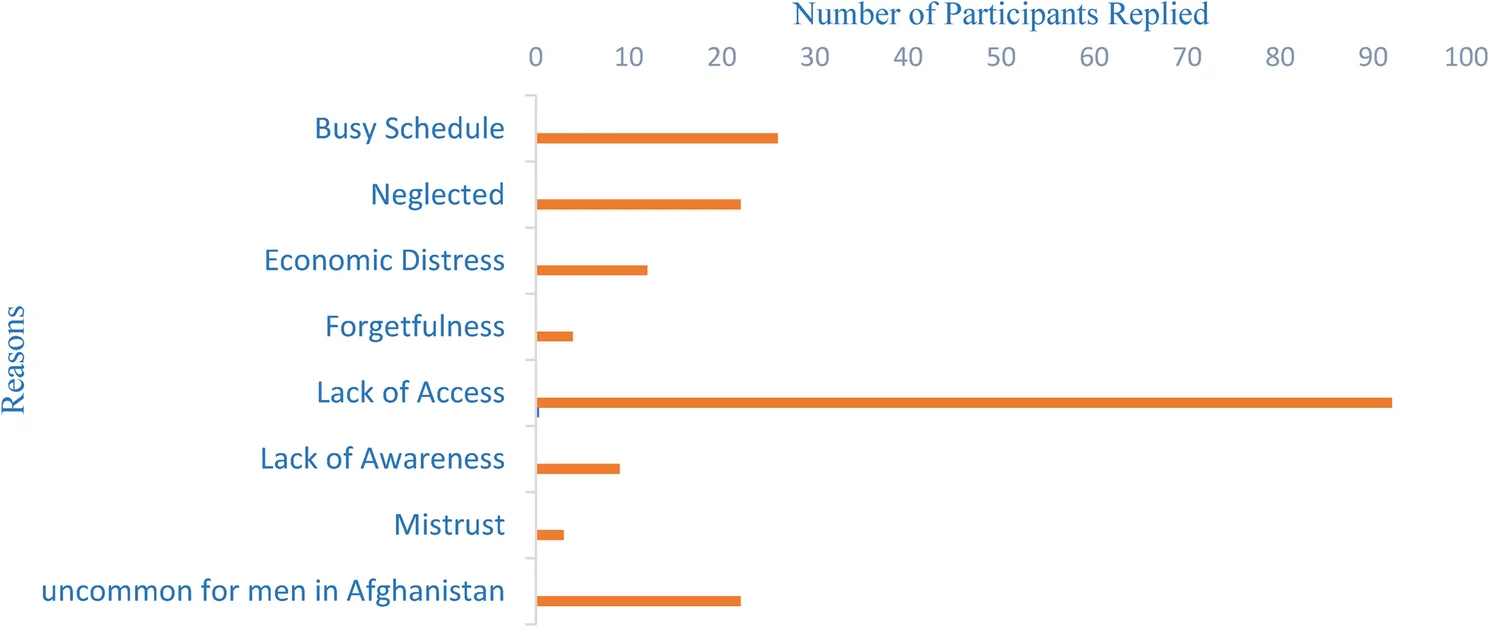
Self-reported reasons for non-vaccination against hepatitis B and tetanus toxoid among healthcare workers at three KUMS-affiliated teaching hospitals in Kabul, Afghanistan (*n* = 190)

This study found that a considerable number of participants did not receive any dose of either vaccine or missed some doses (*n* = 224). Therefore, the participants were asked about the reasons for non-vaccination. Approximately 15.2% (*n* = 34) did not provide a specific reason. Among those who provided a reason (*n* = 190, 84.8%), reasons included lack of access (48.4%), busy schedule (13.7%), neglected to vaccinate (11.6%), a perception that vaccination is uncommon for men in Afghanistan (11.6%), economic distress (6.3%), lack of awareness (4.7%), forgetfulness (2.1%), and mistrust (1.6%), Fig. 1. It is noteworthy, however, that despite the barriers, nearly all participants (99.3%) agreed with and recommended vaccination.

### Multivariable analysis

Tables 4 and 5 present the unadjusted and adjusted odds ratio estimates from the logistic regression analyses identifying factors independently associated with hepatitis B and tetanus vaccination among healthcare workers, respectively. For hepatitis B vaccination, two factors emerged as significant independent predictors in the multivariable model. Healthcare workers with six or more years of total work experience had nearly twice the odds of being vaccinated compared to those with 1–5 years of experience (aOR = 1.85, 95% CI: 1.16–2.96, *p* = 0.02). Regarding profession, midwives and nurses had significantly lower odds of hepatitis B vaccination compared to physicians (aOR = 0.62, 95% CI: 0.39–0.97, *p* = 0.04). No significant associations were found for age, gender, marital status, or monthly income. For tetanus vaccination, gender was the strongest predictor, with females having 20 times higher odds of being vaccinated compared to males (aOR = 20.09, 95% CI: 12.26–32.91, *p* ≤ 0.001). Profession was also significantly associated; midwives and nurses had more than twice the odds of tetanus vaccination compared to physicians (aOR = 2.19, 95% CI: 1.17–4.10, *p* = 0.03). Other health professions showed a trend toward higher odds but did not reach statistical significance (aOR = 1.50, 95% CI: 0.92–2.46, *p* = 0.09). Monthly income categories approaching significance (*p* = 0.09 and *p* = 0.06) suggest a possible association with higher income, though confidence intervals crossed one. Age, marital status, and work experience were not significantly associated with tetanus vaccination.

**Table 4:** Unadjusted and adjusted logistic regression of factors associated with hepatitis B vaccination among healthcare workers at three KUMS-affiliated teaching hospitals in Kabul, Afghanistan (*N* = 284)

	Unadjusted				Adjusted			
	OR	95% CI		*P*-value	aOR	95% CI		*P*-value
Variable		LL	UL			LL	UL	
Age								
19–24 years	Ref.				Ref.			
25–34 years	1.55	0.69	3.49	0.21	1.37	0.47	4.00	0.46
35–44 years	2.90	0.56	15.14	0.15	1.92	0.28	12.98	0.40
45–62 years	2.45	0.79	7.61	0.09	1.58	0.37	6.77	0.43
Gender								
Male	Ref.				Ref.			
Female	1.20	0.50	2.90	0.59	1.59	0.78	3.25	0.14
Marital Status								
Single	Ref.				Ref.			
Married	1.40	0.57	3.44	0.36	0.90	0.27	3.04	0.83
Total Work Experience								
1–5 years	Ref.				Ref.			
≥ 6 years	2.11	1.39	3.21	0.01	1.85	1.16	2.96	0.02
Profession								
Physicians	Ref.				Ref.			
Midwives/Nurses	0.56	0.28	1.12	0.08	0.62	0.39	0.97	0.04
Other Health Professions	0.87	0.81	0.93	0.00	0.87	0.52	1.46	0.49
Monthly Income								
Less than 10,000 AFs	Ref.				Ref.			
10,000–20,000 AFs	0.75	0.41	1.38	0.26	0.79	0.40	1.58	0.40
More than 20,000 AFs	1.17	0.71	1.94	0.43	0.98	0.48	1.99	0.94

*OR* Odds ratio, *aOR* Adjusted odds ratio, *CI* Confidence interval, *LL* lower limit, *UL* Upper limit

*P-*values are based on survey-weighted logistic regression

**Table 5:** Unadjusted and adjusted logistic regression of factors associated with tetanus toxoid vaccination among healthcare workers at three KUMS-affiliated teaching hospitals in Kabul, Afghanistan (*N* = 284)

	Unadjusted				Adjusted			
	OR	95% CI		*P*-value	aOR	95% CI		*P*-value
Variable		LL	UL			LL	UL	
Age								
19–24 years	Ref.				Ref.			
25–34 years	0.62	0.16	2.47	0.40	1.04	0.30	3.60	0.93
35–44 years	0.23	0.02	3.18	0.20	0.83	0.08	9.05	0.84
45–62 years	0.32	0.03	3.83	0.27	1.08	0.13	8.74	0.92
Gender								
Male	Ref.				Ref.			
Female	20.14	6.30	64.36	0.00	20.09	12.26	32.91	≤ 0.001
Marital Status								
Single	Ref.				Ref.			
Married	0.52	0.18	1.50	0.16	0.96	0.50	1.84	0.86
Total Work Experience								
1–5 years	Ref.				Ref.			
≥ 6 years	0.61	0.15	2.54	0.39	1.04	0.44	2.50	0.90
Profession								
Physicians	Ref.				Ref.			
Midwives/Nurses	2.48	0.05	136.12	0.56	2.19	1.17	4.10	0.03
Other Health Professions	0.97	0.01	77.70	0.99	1.50	0.92	2.46	0.09
Monthly Income								
Less than 10,000 AFs	Ref.				Ref.			
10,000–20,000 AFs	1.74	0.56	5.39	0.25	2.12	0.82	5.51	0.09
More than 20,000 AFs	1.20	0.12	11.79	0.84	2.11	0.96	4.61	0.06

*OR* Odds ratio, *aOR* Adjusted odds ratio, *C*I Confidence interval, *LL* Lower limit, *UL* Upper limit

*P-*values are based on survey-weighted logistic regression

## Discussion

Healthcare workers in low-income countries face disproportionate risk of vaccine-preventable occupational infections, yet vaccination coverage in these settings remains poorly characterized. This study is only the second to assess hepatitis B vaccination and the first to examine tetanus vaccination coverage among healthcare workers in Afghanistan, contributing critical evidence to inform occupational health policy in a chronically under-resourced setting. The central finding is that the drivers for vaccination uptake are not uniform but are highly disease-specific, shaped by occupational, institutional, and socio-cultural factors.

Our findings align with the only other study of HBV vaccination among Afghan healthcare workers. Roien et al. (2018–2019) reported that 56.4% had received at least one dose of HBV vaccine, but only 6.8% had completed all three doses [14]. Our finding of 57.8% full vaccination suggests improvement in recent years, though coverage remains far below recommended levels. Our profession-specific rates, 72.6% among physicians, 59.8% among midwives/nurses, and 69.7% among other health professionals, are similar to those reported in Pakistan (64.9%, 75.2%, and 58.3%, respectively), though the higher rate among Pakistani nurses may reflect greater vaccine availability [16]. As comparison, coverage was considerably higher in China (80.8%) and the United States (80.7%) [17, 18]. Notably, several hospitals in the Chinese study offered free HBV vaccination. This type of program has never been implemented at KUMS-affiliated hospitals, underscoring the role of institutional support in achieving high coverage.

For tetanus, our overall coverage of 49.3% receiving at least one dose is comparable to the 50.5% reported among healthcare workers in Somalia [19]. However, our rates were substantially lower than those reported in Turkey, where 76.6% of physicians and 54.4% of nurses had received a tetanus vaccine within the past five years, compared to 26% and 45.1% in our study [20, 21]. Conversely, our coverage was markedly higher than the 2.8% reported among hospital staff in Brazil [22]. These differences likely reflect variation in occupational health infrastructure and vaccine access across settings.

Overall, 67.3% of participants were vaccinated against hepatitis B (partially or fully) and 57.8% had completed the full three-dose series, both figures falling alarmingly short of the near-universal coverage recommended for HCWs who are at high risk of exposure. In the adjusted model, two factors emerged as significant independent predictors of HBV vaccination: profession and work experience. The association between profession and HBV vaccination is striking. Midwives and nurses had significantly lower odds of vaccination compared to physicians. While the reasons for this disparity are not fully clear, it highlights the need for targeted outreach to nursing and midwifery cadres. Although education was not retained in the adjusted model due to collinearity with profession, a strong gradient was observed in the bivariate analysis, with vaccination coverage increasing progressively from 51.1% among Grade 14 graduates to 90.5% among specialists. The association between higher educational attainment and HBV vaccination aligns with the well-established link between health literacy and preventive health behaviors [23, 24], as those with advanced degrees likely have greater awareness of the consequences of HBV infection and the efficacy of the vaccine. The significantly higher vaccination rate among HCWs with six or more years of experience suggests that workplace safety programs have a cumulative effect. Healthcare workers with 1–5 years of experience had lower coverage than those with six or more years, highlighting a potential gap in onboarding processes and ongoing monitoring.

Overall, 49.3% of participants were vaccinated against tetanus (partially or fully), and 45.5% had completed the full five-dose series, revealing a markedly different dynamic from HBV vaccination. The most profound predictor was gender, with female HCWs being 20 times more likely to be vaccinated than males after controlling for other predictors. This almost certainly reflects the benefit of Afghanistan’s public health program to prevent maternal and neonatal tetanus, which integrates tetanus vaccination into antenatal care. Female HCWs of reproductive age, by virtue of having access to the healthcare system, are highly likely to have received the vaccine as part of their own obstetric or gynecological care. This pattern, whereby a successful vertical health program indirectly boosts coverage in a sub-population, is characteristic of tetanus toxoid immunization programs in low-income settings. In contrast, the vast majority of male HCWs (80.7%) were completely unvaccinated, with only 1.3% fully protected, highlighting the absence of any equivalent program targeting men. Profession was also a significant predictor of tetanus vaccination; midwives and nurses had more than twice the odds of vaccination compared to physicians, likely reflecting their closer alignment with maternal and reproductive health services and thus greater exposure to tetanus vaccination through that pathway. Income showed a trend toward higher odds of tetanus vaccination but did not reach statistical significance, suggesting a possible association that warrants further investigation in larger studies. The finding among younger and less experienced workers of higher tetanus coverage may be confounded by age, as younger individuals were more likely to have received tetanus vaccination through maternal health programs.

The reasons cited for non-vaccination provide a clear agenda for intervention. The most significant barrier was lack of access, reported by 48.4% of unvaccinated or partially vaccinated participants, pointing to fundamental health system failures including inconsistent vaccine supply, limited availability of vaccination sites, and cost. These challenges must be understood in the broader context of Afghanistan’s fragile health system, which has faced compounding crises including prolonged conflict, political instability, and the disruption of health services during and after the COVID-19 pandemic. Conflict and political instability have repeatedly disrupted cold chain infrastructure, reduced the health workforce, and curtailed vaccination campaigns, while the COVID-19 pandemic further strained an already under-resourced system and diverted resources away from routine immunization services [25–27]. 

Furthermore, hepatitis B vaccination in public health facilities is available only for children; adults must access private facilities and pay out of pocket. Tetanus vaccine is an exception, available through public facilities for pregnant women. The fact that 66% of HBV vaccines were administered in private facilities further illustrates the failure of the public system to provide this essential occupational health service. Despite these barriers, nearly all participants (99.3%) held positive beliefs about vaccines. This confirms that the primary challenge is not vaccine hesitancy but rather systemic and logistical barriers to access, a distinction with important implications for intervention design.

Regarding the broader risk implications of low vaccination coverage, unvaccinated HCWs with direct patient contact face documented occupational risk of HBV acquisition, particularly in highly endemic settings where general population prevalence amplifies exposure risk [28]. Critically, the risk is bidirectional: a recent systematic review identified confirmed HBV transmissions from healthcare workers to patients across multiple countries, underscoring that unvaccinated HCWs represent a preventable transmission risk within healthcare settings [29]. Afghanistan-specific iatrogenic transmission data are limited, but the global evidence reinforces the urgency of institutional action, particularly given the well-documented decline in vaccination program capacity during and after the COVID-19 pandemic [30]. 

These findings point to a clear and actionable policy gap: the absence of a formal occupational vaccination program for healthcare workers in Afghanistan. Institutional interventions, including employer-provided vaccination, integration of HBV and tetanus vaccination into onboarding processes, and removal of cost barriers, are essential steps toward protecting both healthcare workers and the patients they serve. KUMS-affiliated teaching hospitals, as the primary training centers for Afghanistan’s health workforce, are uniquely positioned to lead by example in implementing such programs.

### Limitations

This study has several limitations. Its cross-sectional design can establish associations but not causality. Vaccination status and reasons for non-vaccination were self-reported rather than confirmed by serological testing or medical record review, and actual immunity may differ from reported coverage. Participants may have over-reported vaccination or understated vaccine hesitancy given professional expectations, potentially leading to an overestimation of coverage due to social desirability bias. The study was conducted in three teaching hospitals in Kabul, which may limit generalizability to HCWs in private clinics or rural settings across Afghanistan. Work experience was captured in two categories (1–5 years and ≥ 6 years) based on the distribution of the data, which limits the ability to examine finer gradations of experience. Full tetanus toxoid vaccination was defined as receipt of five doses, consistent with WHO recommendations for prevention of maternal and neonatal tetanus and Afghanistan’s national immunization program; however, as this schedule is specifically designed for women of reproductive age, applying this threshold uniformly to male participants may underestimate adequate tetanus protection among men, and tetanus coverage findings should be interpreted accordingly.

### Conclusion and recommendations

In conclusion, this study reveals that vaccination coverage among healthcare workers in three teaching hospitals in Kabul is influenced by distinct factors. Hepatitis B vaccination is influenced primarily by occupational role and work experience. In contrast, tetanus vaccination is predominantly shaped by gender and income, reflecting the indirect benefit of maternal health programs and the role of personal resources.

The critical barrier to vaccination is not hesitancy, as evidenced by near-universal positive attitudes, but rather systemic failure. The predominant issue of “lack of access,” coupled with heavy reliance on private facilities for HBV vaccines, highlights a significant public health system shortfall in providing essential occupational health services.

To address this, we recommend a multi-pronged strategy:

1. Strengthen Institutional Vaccination Programs: Ensure vaccine availability and implement mandatory, documented HBV vaccination as a condition of employment for all HCWs, with a particular focus on new recruits and physicians. The public health system must ensure reliable, free, and on-site access to vaccines to eliminate the reliance on private facilities.
2. Leverage Successes: The high tetanus vaccination coverage in women demonstrates the effectiveness of integrated, accessible programs. Similar models of easy access and integration into routine health activities should be explored for other vaccines, including offering tetanus vaccination boosters to male HCWs who are at occupational risk.
3. Address Specific Barriers: Targeted interventions are needed to combat “lack of access” and “busy schedules.” This could include setting up vaccination stations in public hospitals, dedicating specific “vaccination hours” for staff, and implementing robust reminder systems.
4. Capitalize on Positive Attitudes: Launch internal communication campaigns that leverage the near-universal pro-vaccination attitude among staff to create a culture where being fully vaccinated is the professional and social norm.

## Supplementary Information

Supplementary Material 1.

## Data Availability

Research data are generally not shared per the institutional policies of Kabul University of Medical Sciences, but reasonable requests will be considered by contacting the corresponding author.

## Abbreviations

HBV: Hepatitis B Virus
HCW: Healthcare worker
KUMS: Kabul University of Medical Sciences
TT: Tetanus Toxoid
