# Supplementary material for: Assessment of hepatitis B and tetanus toxoid vaccination coverage and associated factors among healthcare workers at Kabul University of Medical Sciences affiliated hospitals in Afghanistan
Source: BMC Public Health. 2026 Jul 3;26:2668. doi: 10.1186/s12889-026-27775-6 (PMC13602782; doi:10.1186/s12889-026-27775-6)
Supplement: Supplementary file 1 [file 12889_2026_27775_MOESM1_ESM.docx]

**Questionnaire**

Assessment of Hepatitis B and Tetanus Vaccination Coverage and Associated Factors Among Healthcare Providers Working at the Kabul University of Medical Sciences Affiliated Hospitals in Afghanistan

1. Age: _________________________________

2. Gender: _________________________________

3. Marital status: Single ________	Married ________	Widowed ________

4. Educational level: _________________________________

5. Work experience: _________________________________ years

6. Occupation: _________________________________

7. Department/Unit: _________________________________

8. Monthly income: _________________________________

9. Family history of hepatitis: _________________________________

10. Family history of Tetanus: _________________________________

11. Personal beliefs or cultural practices regarding vaccination: _________________________________

12. Date of first hepatitis B vaccination dose received: ______ / ______ / ______

13. Number of doses received, date(s), and location(s) of administration: ______ / ______ / ______

14. Date of first Tetanus dose received: ______ / ______ / ______

15. Number of Tetanus doses received, date(s), and location(s) of administration: ______ / ______ / ______

16. Reasons for not receiving the vaccine: _________________________________

17. Other vaccines received: _________________________________

18. Exposure to patients with Hepatitis B: _________________________________

19. Exposure to patients with Tetanus: _________________________________
